# Stress monitoring capability of magnetostrictive Fe–Co fiber/glass fiber reinforced polymer composites under four-point bending

**DOI:** 10.1038/s41598-022-25792-0

**Published:** 2022-12-27

**Authors:** Kenichi Katabira, Tomoki Miyashita, Fumio Narita

**Affiliations:** 1 Department of Materials Processing, Graduate School of Engineering, Tohoku University, Aoba-Yama 6-6-02, Sendai, 980-8579 Japan; 2 Department of Frontier Sciences for Advanced Environment, Graduate School of Environmental Studies, Tohoku University, Aoba-Yama 6-6-02, Sendai, 980-8579 Japan

**Keywords:** Sensors and biosensors, Characterization and analytical techniques, Composites

## Abstract

Many structural health monitoring (SHM) techniques have been investigated for damage detection in woven glass fiber reinforced polymer (GFRP) laminates. Recently, the GFRP composites integrated with sensors have received attention because the composite material can transmit information about the structural condition during operation. Magnetostrictive materials are considered as feasible candidates to realize the contactless SHM techniques by exploiting the Villari effect, but the theoretical modeling to correlate a magnetostrictive response with structural conditions is a critical issue. In this study, the analytical procedure considering the mechanics of materials and electromagnetism was proposed to model the magnetic induction by the Villari effect of magnetostrictive GFRP laminates under bending. The magnetostrictive Fe–Co fiber/GFRP composites were then developed, and the four-point bending tests were carried out to evaluate the fabricated composites’ stress monitoring capability. The magnetic flux density behavior corresponded to the bending stress fluctuation. The maximum magnetic flux density change was 70.7 mT subjected to the peak bending stress of 158 MPa. The analytical solutions showed reasonable agreement with the experimental results. The applied stress and measured magnetic flux density were correlated by the theoretical models. Thus, these results suggest an important step in realizing the novel contactless SHM technique utilizing magnetostrictive materials.

## Introduction

Woven glass fiber reinforced polymer (GFRP) laminates exhibit the features of thermal insulation, electrical insulation, and excellent mechanical properties, and are good materials for superconducting devices for use in the fusion reactor, such as the International Thermonuclear Experimental Reactor (ITER)^1^. However, the application of FRP laminates is prone to be limited due to their complex damage and failure morphologies, for example, interlaminar failure^2,3^. Hence, it has been required to assess the damage state and predict the remaining service life for safe operation^4^.

Structural health monitoring (SHM) is required to maintain safety protocols for these structural components during service^5^. Many researchers have studied various SHM techniques, for instance, the frequency method^6^, Lamb waves^7^, and acoustic emission^8^. However, a versatile technique for all conditions, situations, and applications has not been created because every developed technique has their own advantages, limitations, and scope of application^9^. Currently, the composites embedded with sensors have been broadly recognized as one of the SHM technologies since the composite material can inform the structural health by itself. Fiber optic sensors in composite structures have received attention due to their distinctive advantages^10^. Sánchez et al.^11^ have monitored the full manufacturing process of carbon fiber reinforced polymer (CFRP) embedded with optical fiber sensors and evaluated the distributed residual strain profile. The GFRP feasibility with optical backscatter reflectometer based on Rayleigh scatter has been explored^12^. Okabe et al.^13^ have demonstrated that the sensing capability of chirped fiber Bragg grating to identify crack locations in CFRP laminates. Electrical resistance measurement has been investigated since the damage and the electrical resistance in CFRP composites can be coupled^14^. The impact damage on continuous carbon fiber/epoxy composite laminates has been evaluated by electrical resistance measurement, and the sensitivity of the technique was more effective than that of ultrasonic methods^15^. The correlation between the interlaminar shear behavior and electrical resistance responses of woven CFRP composite laminates in a cryogenic environment has been discussed numerically and experimentally^16,17^. Takeda and Narita^18^ have reported the crack propagation monitoring of bonded CFRP composite joints with carbon nanotube/epoxy adhesive layer under Mode I loading. Piezoelectric materials can be utilized as both passive and active sensors bonded to a composite structure^19^. CFRP composites embedded with piezoelectric ceramics have been characterized to discuss the real-time SHM ability^20,21^. Hwang et al.^22^ have characterized a piezoelectric GFRP composite laminate including a mixture of piezoelectric powder and epoxy resin for an impact sensor. Smart woven fiber reinforced polymer (FRP) composite laminates composed of woven piezoelectric fabric, which acts as a sensor and a reinforcement, have exhibited a direct relationship between the applied load and the sensor signal^23^. Wang et al.^24^ have suggested the novel polarization process of the piezoelectric CFRP composites and characterized the piezoelectric property.

**Figure 1:**
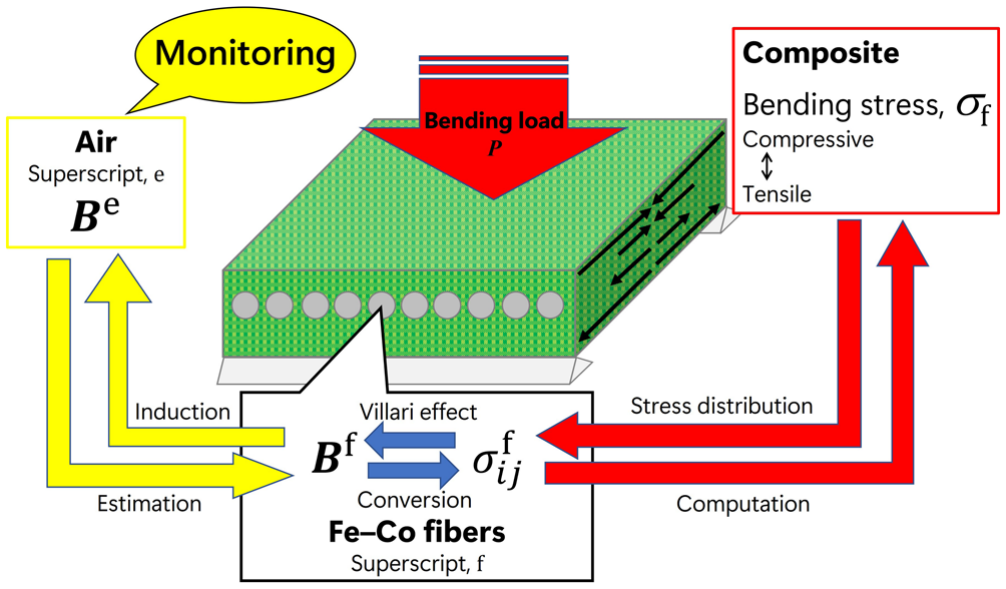
Magnetostrictive behavior and stress monitoring procedure of Fe–Co fiber/GFRP composite under bending load.

Magnetostrictive materials have been employed for sensor or energy harvesting applications by exploiting the Villari effect, which is generally described as the change in magnetization of ferromagnetic materials when subjected to applied stress^25^. Hence, unlike other composites embedded with SHM sensor, magnetostrictive FRP composites are expected to realize the FRP structures’ contactless monitoring because the magnetic field change caused by magnetostrictive materials can be picked up as voltage change using a coil or a Hall probe. Terfenol-D (Tb_1−*x*_Dy_*x*_Fe_2_) is known as a giant magnetostrictive material. Kubicka et al.^26,27^ have reported the preparation and characterization of CFRP embedded with Terfenol-D particles. Additionally, other researchers have studied magnetostrictive polymer composites for SHM applications^28–31^. Fe–Co alloy (Fe_29_Co_71_) exhibits ductility and good workability; therefore, Fe–Co wires have been implanted in epoxy resin to invent original magnetostrictive epoxy composite materials utilizing the high aspect ratio of Fe–Co wires^32–35^. The studies have mainly investigated the magnetostrictive response to compressive stress. Lately, Katabira et al.^36,37^ have developed FRP composites embedded with Fe–Co wires and investigated the effects of the composite design on the self-sensing capability under bending loading. The composite design using Fe–Co wire is easy to control the magnetization direction because of the strong columnar crystals, and this feature has possibility to eliminate the need for application of a bias magnetic field. Figure 1 shows the magnetostrictive behavior and stress monitoring procedure of GFRP composite embedded with Fe–Co fibers (Fe–Co fiber/GFRP composite) under bending load *P*. Bending stress $\sigma_{{\text{f}}}$ generates stress tensor $\sigma_{ij}^{{\text{f}}}$ in Fe–Co fibers, which induces magnetic flux density ${\varvec{B}}^{{\text{f}}}$ by the Villari effect. A magnetic field is then induced around the composite, which is expressed as magnetic flux density ${\varvec{B}}^{{\text{e}}}$ in the air in Fig. 1. The superscripts f and e represent the quantities inside and outside the Fe–Co fibers, respectively. One of the critical issues of the contactless assessment is estimating magnetic flux density in Fe–Co fibers from monitored magnetic flux density around magnetostrictive FRP composites. The correlation between magnetic responses and structural conditions must also be considered based on a theoretical model of the composite structure. However, literature studies about these issues are sparse and inconclusive.

In this study, an analytical procedure based on the mechanics of materials and electromagnetism was proposed to devise an approach to understand the correlation between magnetostrictive responses and structural conditions under bending. Then, the Fe–Co fiber/GFRP composites were fabricated. Four-point bending tests were conducted to investigate the stress self-sensing capability, utilizing the Villari effect of the magnetostrictive GFRP composites. Three Hall probes were introduced to evaluate the magnetic field distribution’s change around the specimens during four-point bending tests.

## Analysis

The basic equations for magnetostrictive material are outlined here. Consider the coordinate system o-*xyz*. The *z*-axis coincides with the easy axis of the magnetization. The constitutive equations are given by

1 $$\left\{ {\begin{array}{*{20}c} {\varepsilon_{xx}^{{\text{f}}} } \\ {\varepsilon_{yy}^{{\text{f}}} } \\ {\varepsilon_{zz}^{{\text{f}}} } \\ {2\varepsilon_{yz}^{{\text{f}}} } \\ {2\varepsilon_{zx}^{{\text{f}}} } \\ {2\varepsilon_{xy}^{{\text{f}}} } \\ \end{array} } \right\} = \left[ {\begin{array}{*{20}c} {s_{11}^{{\text{f}}} } & {s_{12}^{{\text{f}}} } & {s_{13}^{{\text{f}}} } & 0 & 0 & 0 \\ {s_{12}^{{\text{f}}} } & {s_{11}^{{\text{f}}} } & {s_{13}^{{\text{f}}} } & 0 & 0 & 0 \\ {s_{13}^{{\text{f}}} } & {s_{13}^{{\text{f}}} } & {s_{33}^{{\text{f}}} } & 0 & 0 & 0 \\ 0 & 0 & 0 & {s_{44}^{{\text{f}}} } & 0 & 0 \\ 0 & 0 & 0 & 0 & {s_{44}^{{\text{f}}} } & 0 \\ 0 & 0 & 0 & 0 & 0 & {s_{66}^{{\text{f}}} } \\ \end{array} } \right]\left\{ {\begin{array}{*{20}c} {\sigma_{xx}^{{\text{f}}} } \\ {\sigma_{yy}^{{\text{f}}} } \\ {\sigma_{zz}^{{\text{f}}} } \\ {\sigma_{yz}^{{\text{f}}} } \\ {\sigma_{zx}^{{\text{f}}} } \\ {\sigma_{xy}^{{\text{f}}} } \\ \end{array} } \right\} + \left[ {\begin{array}{*{20}c} 0 & 0 & {d_{31}^{{{\prime }{\text{f}}}} } \\ 0 & 0 & {d_{31}^{{{\prime }{\text{f}}}} } \\ 0 & 0 & {d_{33}^{{{\prime }{\text{f}}}} } \\ 0 & {d_{15}^{{{\prime }{\text{f}}}} } & 0 \\ {d_{15}^{{{\prime }{\text{f}}}} } & 0 & 0 \\ 0 & 0 & 0 \\ \end{array} } \right]\left\{ {\begin{array}{*{20}c} {H_{x}^{{\text{f}}} } \\ {H_{y}^{{\text{f}}} } \\ {H_{z}^{{\text{f}}} } \\ \end{array} } \right\},$$

2 $$\left\{ {\begin{array}{*{20}c} {B_{x}^{{\text{f}}} } \\ {B_{y}^{{\text{f}}} } \\ {B_{z}^{{\text{f}}} } \\ \end{array} } \right\} = \left[ {\begin{array}{*{20}c} 0 & 0 & 0 & 0 & {d_{15}^{{{\prime }{\text{f}}}} } & 0 \\ 0 & 0 & 0 & {d_{15}^{{{\prime }{\text{f}}}} } & 0 & 0 \\ {d_{31}^{{{\prime }{\text{f}}}} } & {d_{31}^{{{\prime }{\text{f}}}} } & {d_{33}^{{{\prime }{\text{f}}}} } & 0 & 0 & 0 \\ \end{array} } \right]\left\{ {\begin{array}{*{20}c} {\sigma_{xx}^{{\text{f}}} } \\ {\sigma_{yy}^{{\text{f}}} } \\ {\sigma_{zz}^{{\text{f}}} } \\ {\sigma_{yz}^{{\text{f}}} } \\ {\sigma_{zx}^{{\text{f}}} } \\ {\sigma_{xy}^{{\text{f}}} } \\ \end{array} } \right\} + \left[ {\begin{array}{*{20}c} {\mu_{11}^{{\text{f}}} } & 0 & 0 \\ 0 & {\mu_{11}^{{\text{f}}} } & 0 \\ 0 & 0 & {\mu_{33}^{{\text{f}}} } \\ \end{array} } \right]\left\{ {\begin{array}{*{20}c} {H_{x}^{{\text{f}}} } \\ {H_{y}^{{\text{f}}} } \\ {H_{z}^{{\text{f}}} } \\ \end{array} } \right\},$$

where $\varepsilon_{xx}^{{\text{f}}}$, $\varepsilon_{yy}^{{\text{f}}}$, $\varepsilon_{zz}^{{\text{f}}}$, $\varepsilon_{yz}^{{\text{f}}} = \varepsilon_{zy}^{{\text{f}}}$, $\varepsilon_{zx}^{{\text{f}}} = \varepsilon_{xz}^{{\text{f}}}$, $\varepsilon_{xy}^{{\text{f}}} = \varepsilon_{yx}^{{\text{f}}}$ are the components of the strain tensor, $\sigma_{xx}^{{\text{f}}}$, $\sigma_{yy}^{{\text{f}}}$, $\sigma_{zz}^{{\text{f}}}$, $\sigma_{yz}^{{\text{f}}} = \sigma_{zy}^{{\text{f}}}$, $\sigma_{zx}^{{\text{f}}} = \sigma_{xz}^{{\text{f}}}$, $\sigma_{xy}^{{\text{f}}} = \sigma_{yx}^{{\text{f}}}$ are the components of the stress tensor, $H_{x}^{{\text{f}}}$, $H_{y}^{{\text{f}}}$, and $H_{z}^{{\text{f}}}$ are the components of the magnetic field intensity vector, $B_{x}^{{\text{f}}}$, $B_{y}^{{\text{f}}}$, and $B_{z}^{{\text{f}}}$ are the components of the magnetic flux density vector, $s_{11}^{{\text{f}}}$, $s_{33}^{{\text{f}}}$, $s_{44}^{{\text{f}}}$, $s_{66}^{{\text{f}}}$, $s_{12}^{{\text{f}}}$, $s_{13}^{{\text{f}}}$ are the elastic compliances at constant magnetic field, $d_{15}^{{\prime}{\text{f}}}$, $d_{31}^{{\prime}{\text{f}}}$, and $d_{33}^{{\prime}{\text{f}}}$ are the magnetoelastic constants, and $\mu_{11}^{{\text{f}}}$ and $\mu_{33}^{{\text{f}}}$ are the magnetic permeabilities at constant stress, respectively. Usually, Fe–Co fiber is manufactured by drawing. It is assumed that the easy axis of the magnetization is along the length direction and that the longitudinal (33) magnetostrictive deformation mode is dominant. Hence, the constants $d_{15}^{{\prime}{\text{f}}}$, $d_{31}^{{\prime}{\text{f}}}$, and $d_{33}^{{\prime}{\text{f}}}$ are

3 $$d_{15}^{{{\prime }{\text{f}}}} { } = { }d_{15}^{{\text{f}}} , d_{31}^{{{\prime }{\text{f}}}} = d_{31}^{{\text{f}}} , d_{33}^{{{\prime }{\text{f}}}} = d_{33}^{{\text{f}}} + m_{33}^{{\text{f}}} H_{z} ,$$

where $d_{15}^{{\text{f}}}$, $d_{31}^{{\text{f}}}$, and $d_{33}^{{\text{f}}}$ are the piezomagnetic constants, and $m_{33}^{{\text{f}}}$ is the second-order magnetoelastic constant. Here, we focus on the Fe–Co fiber and consider a one-dimensional model because the magnetostrictive behavior in the *z*-direction is dominant. The constitutive Eqs. (1) and (2) of Fe–Co fiber become

4 $$\varepsilon_{zz}^{{\text{f}}} = { }s_{33}^{{\text{f}}} \sigma_{zz}^{{\text{f}}} ,$$

5 $$B_{z}^{{\text{f}}} = d_{33}^{{{\prime }{\text{f}}}} \sigma_{zz}^{{\text{f}}} .$$

Here, it is assumed that the component of the magnetic field intensity vector is omitted because a bias magnetic field was not applied on the specimen^38^.

**Figure 2:**
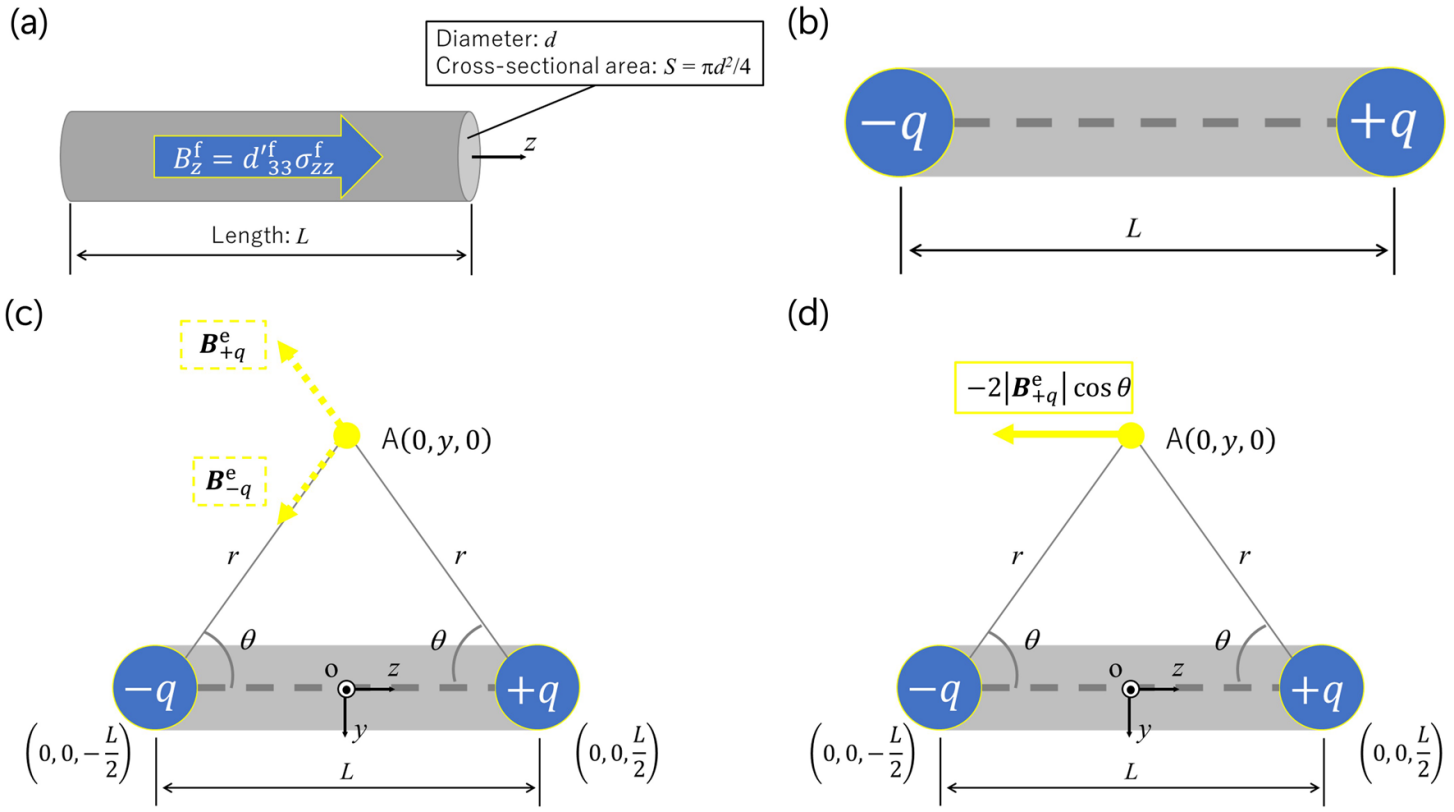
(**a**) The Fe–Co fiber and the magnetic flux density, (**b**) the magnetic charges, (**c**) magnetic flux density induced by two magnetic charges at point A, and (**d**) *z*-component of the magnetic flux density induced by magnetic charges at point A.

Figure 2a shows the Fe–Co fiber of length *L* and diameter *d*. The magnetic flux density is induced due to the normal stress along the length (*z*-)direction (easy axis). Then, the Fe–Co fiber (Fig. 2a) is assumed to have two magnetic charges, ± *q*, at both ends (Fig. 2b). The magnetic charge due to the Villari effect is assumed to be equal to the amount of the magnetic flux in the *z*-direction ${\phi_{z}^{\text{f}}}$ through the magnetostrictive fiber’s cross-section, i.e.,

6 $$q = {\phi_{z}^{{\text{f}}}} = B_{z}^{{\text{f}}} S = d_{\text{33}}^{{\prime}{\text{f}}} \sigma_{zz}^{{\text{f}}} S,$$

where *S* = π*d*^2^/4 is the cross-sectional area of the Fe–Co fiber. Let us now consider the coordinate system, o-*xyz*, as shown in Fig. 2c, whose origin is positioned at the center of the magnetic charges. The magnetic flux densities at the arbitrary point A(0, *y*, 0) in space induced by magnetic charge + *q* and − *q* are, respectively, given by:

7 $${\varvec{B}}_{ + q}^{{\text{e}}} = \frac{1}{4\pi }\frac{q}{{r^{3} }}\left(0, y, -\frac{L}{2}\right), {\varvec{B}}_{ - q}^{{\text{e}}} = - \frac{1}{4\pi }\frac{q}{{r^{3} }}\left(0, y, \frac{L}{2}\right),$$

where *r* is the distance between the magnetic charge and the arbitrary point A. Here, *z*-component of the magnetic flux density at the arbitrary point A(0, *y*, 0), shown in Fig. 2d, can be expressed as:

8 $$B_{z}^{{\text{e}}} \left( {0, y,0} \right) = \left|{\varvec{B}}_{ + q}^{{\text{e}}}\right| \sin \theta_{+q} +  \left|{\varvec{B}}_{ - q}^{{\text{e}}}\right| \sin \theta_{-q} = -2\left| {{\varvec{B}}_{ + q}^{{\text{e}}} } \right|\cos \theta ,$$

where

9 $$\sin \theta_{+q} = \sin \left( \frac{3}{2}\pi-\theta \right), \sin \theta_{-q} = \sin \left( \frac{3}{2}\pi+\theta \right), \sin \theta_{+q} = \sin \theta_{-q} = - \cos \theta = -\frac{{{\raise0.7ex\hbox{$L$} \!\mathord{\left/ {\vphantom {L 2}}\right.\kern-\nulldelimiterspace} \!\lower0.7ex\hbox{$2$}}}}{r} = -\frac{L}{{2\sqrt {y_{{}}^{2} + \left( \frac{L}{2} \right)^{2} } }}.$$

Therefore, we have

10 $$B_{z}^{{\text{e}}} \left( {0,y,0} \right) = -\frac{qL}{{4\pi \left\{ {y^{2} + \left( \frac{L}{2} \right)^{2} } \right\}^{\frac{3}{2}} }} = -\frac{{B_{z}^{{\text{f}}} SL}}{{4\pi \left\{ {y_{{}}^{2} + \left( \frac{L}{2} \right)^{2} } \right\}^{\frac{3}{2}} }} = -\frac{{d_{33}^{{\prime}{\text{f}}} \sigma_{zz}^{{\text{f}}} d^{2} L}}{{16\left\{ {y_{{}}^{2} + \left( \frac{L}{2} \right)^{2} } \right\}^{\frac{3}{2}} }},$$

From Eq. (10), the magnetic flux density $B_{z}^{{\text{f}}}$ in the Fe–Co fiber will be estimated by the measurement of magnetic flux density $B_{z}^{{\text{e}}} \left( {0,y,0} \right)$. Here, it is assumed that the estimated magnetic flux density uniformly distributes between the magnetic charges. The value of $B_{z}^{{\text{e}}}$ changes with an external load. Hence, from the above analysis, we can predict the stress in Fe–Co fiber by monitoring the magnetic induction’s variation through the magnetostrictive fiber.

**Figure 3:**
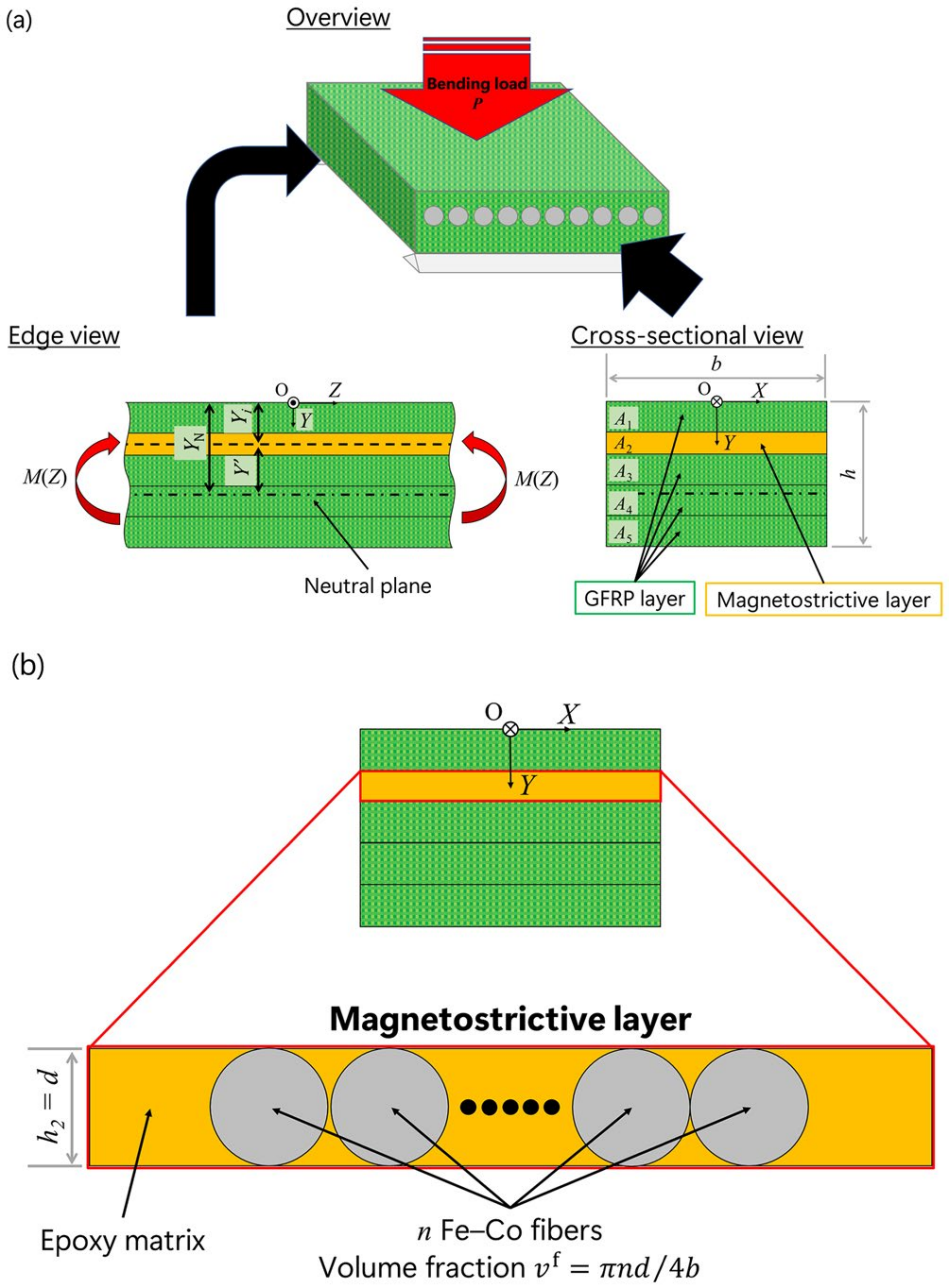
Image of a five layered composite beam with four GFRP layers and one magnetostrictive layer; (**a**) overview, edge view, and cross-sectional view, and (**b**) magnetostrictive layer.

Next, we consider a simply supported five layered composite beam of thickness *h,* width *b* with four GFRP layers, and one magnetostrictive layer under bending moment *M*(*Z*) as shown in Fig. 3a. The origin of the global coordinate system, O-*XYZ*, is at the center of the upper surface of the composite beam, the *X*-axis is in the width direction, and the *Y*- and *Z*-axes are in the thickness and length direction, respectively. The bending moment *M*(*Z*) is induced by the bending load, *P*. For simplicity, it was assumed that the magnetostrictive layer consists of *n* Fe–Co fibers and epoxy matrix; where *n* is the number of Fe–Co fibers. The magnetostrictive layer is the second layer of the composite beam. The position of the neutral plane *Y*_N_ is not the center of the composite beam because of the asymmetric structure, which can be expressed as:

11 $$Y_{{\text{N}}} = \frac{{\mathop \sum \nolimits_{i = 1}^{5} \left( {E_{33} } \right)_{i} \mathop \smallint \nolimits_{{A_{i} }}^{{}} YdA}}{{\mathop \sum \nolimits_{i = 1}^{5} A_{i} \left( {E_{33} } \right)_{i} }},$$

where (*E*_33_)_*i*_ and *A*_*i*_ = *bh*_*i*_ are the Young’s modulus and cross-sectional area of the *i*th layer, respectively, and *h*_*i*_ is the thickness of the *i*th layer. The Young’s moduli of the magnetostrictive layer and GFRP layer are (*E*_33_)_2_ = 1/$s_{33}^{{\text{M}}}$ and (*E*_33_)_1_ = (*E*_33_)_3_ = (*E*_33_)_4_ = (*E*_33_)_5_ = 1/$s_{33}^{{\text{G}}}$, respectively. The superscripts M and G denote the magnetostrictive layer and GFRP layer, respectively. If the distance between the neutral plane and the center plane of the *j*th layer is *Y*′ = *Y*_*j*_ − *Y*_N_, the normal stress in the *j*th layer can be expressed as:

12 $$\left( {\sigma_{ZZ} } \right)_{j} = \frac{{\left( {E_{33} } \right)_{j} M\left( Z \right)Y^{\prime}}}{{\mathop \sum \nolimits_{i = 1}^{5} \left( {E_{33} } \right)_{i} I_{i} }},$$

where the moment of inertia of cross-sectional area of *i*th layer is given by:

13 $$I_{i} = \mathop \int \limits_{{A_{i} }}^{{}} Y^{{\prime}{2}} dA.$$

Especially, the stress in the magnetostrictive layer is obtained as:

14 $$\left( {\sigma_{ZZ} } \right)_{2} = \frac{{\left( {E_{33} } \right)_{2} M\left( Z \right)\left( {Y_{2} - Y_{{\text{N}}} } \right)}}{{\mathop \sum \nolimits_{i = 1}^{5} \left( {E_{33} } \right)_{i} I_{i} }} = \frac{{M\left( Z \right)\left( {Y_{2} - Y_{{\text{N}}} } \right)}}{{s_{33}^{{\text{M}}} \mathop \sum \nolimits_{i = 1}^{5} \left( {E_{33} } \right)_{i} I_{i} }}.$$

We focus on the magnetostrictive layer as shown in Fig. 3b. The strain tensor component $\varepsilon_{ZZ}^{{\text{m}}}$ for the epoxy matrix is given by

15 $$\varepsilon_{ZZ}^{{\text{m}}} = s_{33}^{{\text{m}}} \sigma_{ZZ}^{{\text{m}}} ,$$

where $\sigma_{ZZ}^{{\text{m}}}$ is the stress tensor component, and $s_{33}^{{\text{m}}}$ is the elastic compliance of the epoxy matrix. The superscript m denotes the epoxy matrix. When Fe–Co fiber and epoxy matrix are perfectly bonded, the strain of the magnetostrictive fiber is:

16 $$\varepsilon_{ZZ}^{{\text{f}}} = \varepsilon_{ZZ}^{{\text{m}}} .$$

Hence, the average stress $\sigma_{ZZ}^{0}$ acting on the cross-sectional area of the magnetostrictive layer can be given by

17 $$\sigma_{ZZ}^{0} = \sigma_{ZZ}^{{\text{f}}} v^{{\text{f}}} + \sigma_{ZZ}^{{\text{m}}} \left( {1 - v^{{\text{f}}} } \right),$$

where *v*^f^ = *n*π*d*/4*b* is the volume fraction of the Fe–Co fiber. In this condition, the Young’s modulus of the magnetostrictive layer is $\left( {E_{33} } \right)_{2} = v^{{\text{f}}} /s_{33}^{{\text{f}}} + \left( {1 - v^{{\text{f}}} } \right)/s_{33}^{{\text{m}}}$. In the global coordinate system, O-*XYZ*, the origin of the coordinate system o-*xyz* is (0, *Y*_2_, 0), and the *x*-, *y*-, and *z*-axes are parallel to the *X*-, *Y*-, and *Z*-axes, respectively. When the average stress $\sigma_{ZZ}^{0}$ in Eq. (17) is equal to the normal stress $\left( {\sigma_{ZZ} } \right)_{2}$ in Eq. (14) using Eqs. (4), (15), and considering condition (16), the stress acting on the Fe–Co fibers can be obtained as:

18 $$\sigma_{ZZ}^{{\text{f}}} \left( Z \right) = \frac{{s_{33}^{{\text{m}}} }}{{s_{33}^{{\text{m}}} v^{{\text{f}}} + s_{33}^{{\text{f}}} \left( {1 - v^{{\text{f}}} } \right)}}\left( {\sigma_{ZZ} } \right)_{2} = \frac{{M\left( Z \right)\left( {Y_{2} - Y_{{\text{N}}} } \right)}}{{s_{33}^{{\text{f}}} \mathop \sum \nolimits_{i = 1}^{5} \left( {E_{33} } \right)_{i} I_{i} }}.$$

By substitution of Eq. (18) into Eq. (5), the magnetic flux density $B_{Z}^{{\text{f}}}$ in the Fe–Co fiber of the composite beam under bending moment can be calculated, which allows us to correlate the external load to the inverse magnetostrictive response. Table 1 lists the material properties used in this study.

**Table 1:** Material properties.

		*s*_33_ (× 10^–12^ m^2^/N)^✽^	*h*_*i*_ (mm)
GFRP layer		30.8	0.1375 (*i* = 1, 3, 4, 5)
Magnetostrictive layer	Fe–Co fiber	5.50	0.1 (*i* = 2)
	Epoxy	400	

^✽^Here, we have dropped the superscripts G, f, and m.

**Figure 4:**
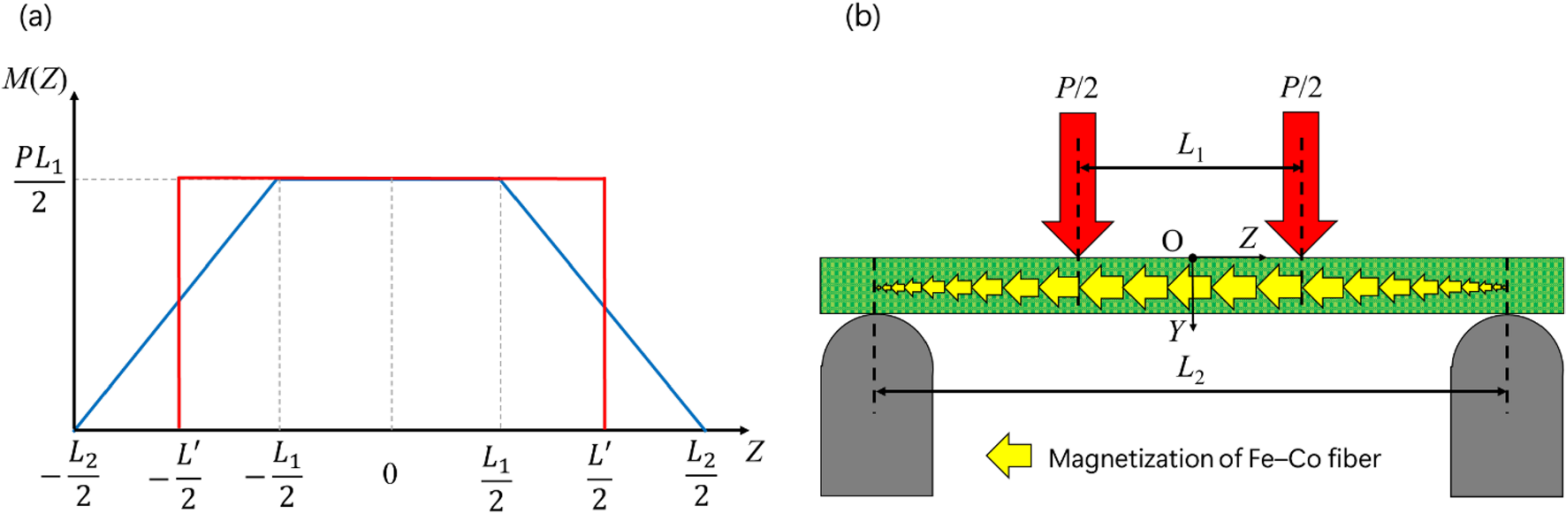
(**a**) Bending moment diagram of four-point bending test, and (**b**) Schematic view of four-point bending test and Fe–Co fiber magnetization.

Finally, the magnetic flux density of Fe–Co fiber under maximum bending stress was discussed. Figure 4a shows the bending moment diagram of four-point bending test, in which the blue line denotes the general bending moment diagram. The bending moment is given by:

19 $$M\left( Z \right) = \left\{ {\begin{array}{*{20}c} {\frac{{PL_{1} }}{{L_{2} - L_{1} }}Z + \frac{{PL_{1} L_{2} }}{{2\left( {L_{2} - L_{1} } \right)}}, - \frac{{L_{2} }}{2} \le Z < - \frac{{L_{1} }}{2}} \\ {\frac{{PL_{1} }}{2}, - \frac{{L_{1} }}{2} \le Z < \frac{{L_{1} }}{2}} \\ { - \frac{{PL_{1} }}{{L_{2} - L_{1} }}Z + \frac{{PL_{1} L_{2} }}{{2\left( {L_{2} - L_{1} } \right)}},\frac{{L_{1} }}{2} \le Z \le \frac{{L_{2} }}{2}} \\ \end{array} } \right..$$

The normal stress for Fe–Co fibers depends on the bending moment. Therefore, the amount of the magnetization of Fe–Co fibers in the specimen is different on the coordinate *Z* as shown in Fig. 4b. To simplify the calculation, the corrected length, *L*′, was then introduced such that the total amount of the magnetization does not change, and the bending moment is constant. In other words, in Fig. 4a, the area surrounded by the red line is equal to the area surrounded by the blue line. Hence, the corrected length was obtained as

20 $$L^{\prime} = \frac{{L_{1} + L_{2} }}{2}.$$

Substituting *L*′ for *L* into Eq. (10), the magnetic flux density of Fe–Co fiber, $B_{Z}^{{\text{f}}}$, was calculated using the experimental values, |*Y*|, $B_{Z}^{{\text{e}}} \left( {0, Y,0} \right)$, and *Y*_A_ = *Y*_2_ – *Y*.

## Experimental procedure

**Figure 5:**
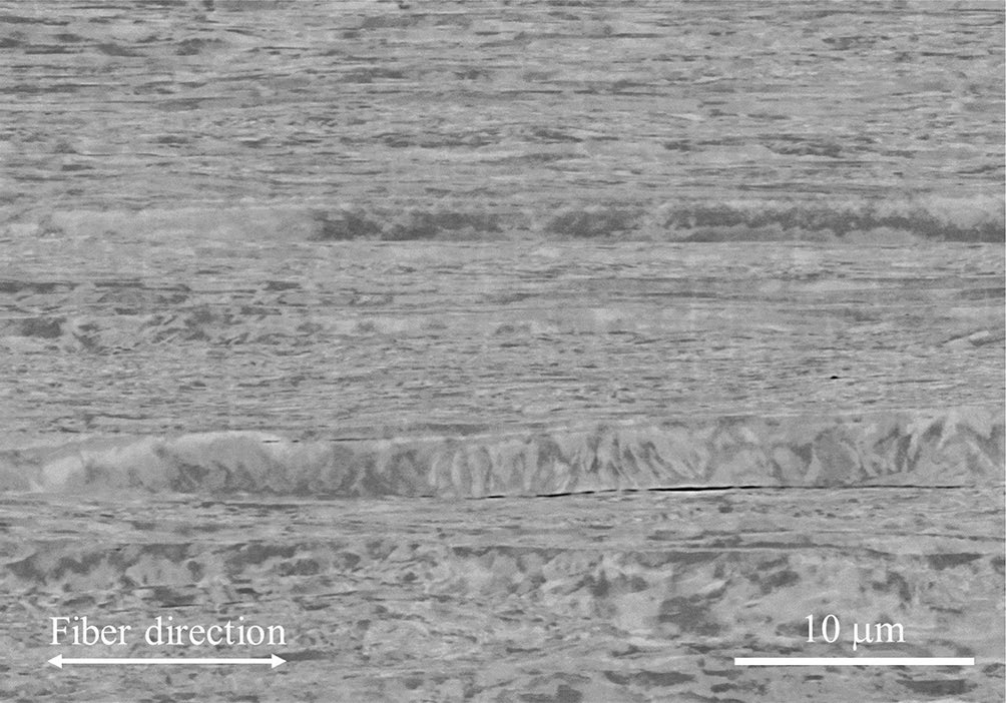
Microstructure of Fe–Co fiber with 100 μm diameters.

**Figure 6:**
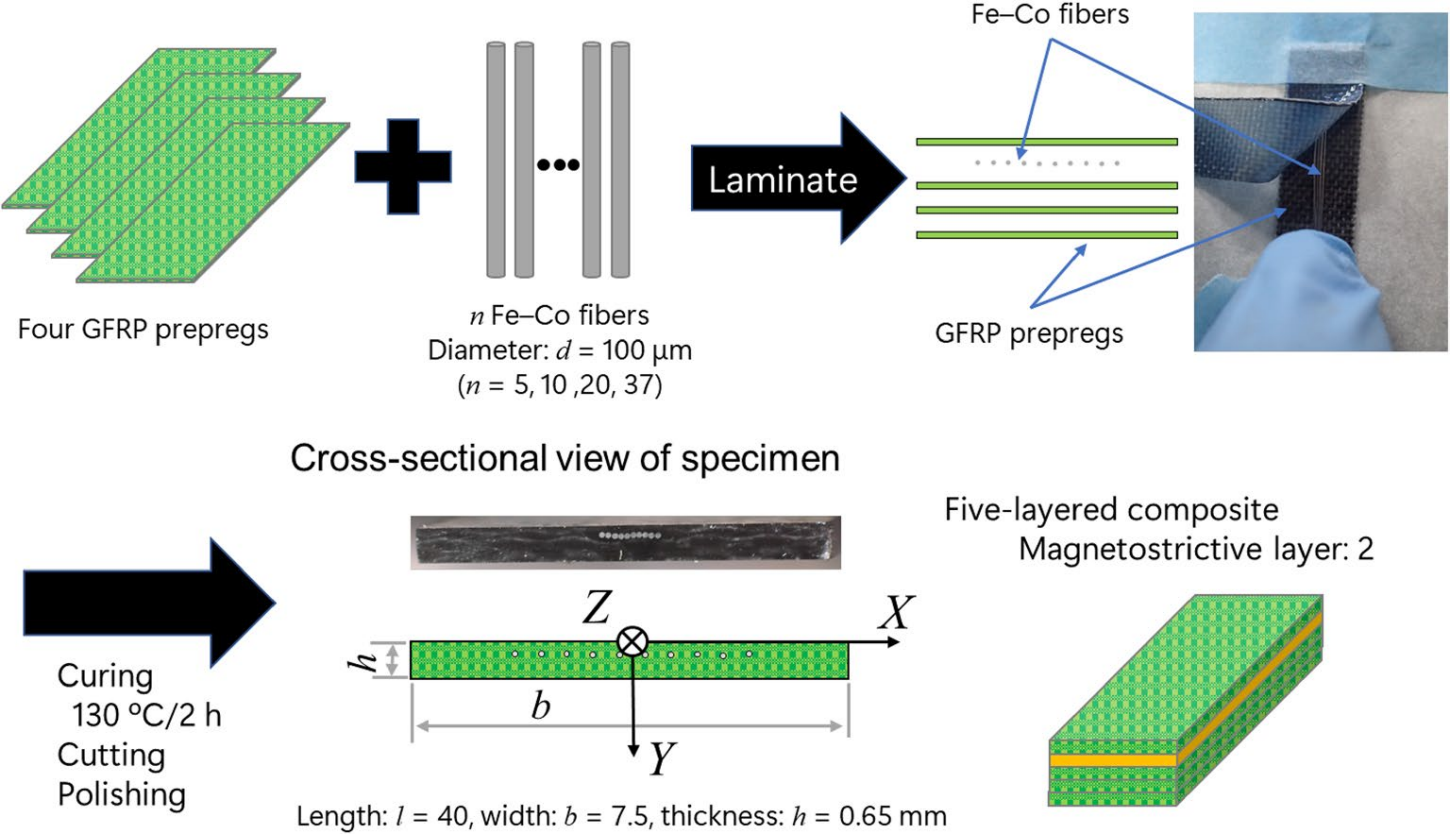
Specimen preparation’s scheme.

The specimens were fabricated using GFRP prepregs (EGP-87 LA18BR, SPIC Corporation, Japan) with a plain weave and magnetostrictive Fe–Co fibers (K-MP70, Tohoku Steel Co. Ltd., Japan) with 100 μm diameters, and the composition of the Fe–Co fibers was Fe_29_Co_71_. Figure 5 shows the microstructure of the Fe–Co fiber. The Fe–Co fiber’s saturation magnetization, *M*_s_, residual magnetization, *M*_r_, and coercivity, *H*_c_, were 1.44 MA/m, 0.31 MA/m, and 6.24 kA/m, respectively. Figure 6 shows the specimen preparation’s scheme. The system of rectangular Cartesian coordinate O-*XYZ* is introduced such that the origin of the system is at the center of the upper surface and the *X*-, *Y*-, and *Z*-axes are along the direction of the specimen’s width, thickness, and length, respectively. Four GFRP prepregs and Fe–Co fibers were laminated followed by curing for 2 h at 130 ºC. The Fe–Co fibers were located on the second layer of the laminate and the distance from the upper surface, *Y*_2_, was 0.1875 mm. The number of Fe–Co fibers, *n*, was 5, 10, 20, and 37. The Fe–Co fibers were closely spaced at the center of the laminate’s width since the measured magnetic flux density will decrease if the Fe–Co fibers are equally spaced. After curing, a laminate was cut and polished so that the specimen’s length, *l*, width, *b*, and thickness, *h*, became 40, 7.5, and 0.65 mm, respectively. The warp direction of GFRP prepreg and the longitudinal direction of Fe–Co fibers are parallel to the *Z*-axis, and the fill direction is parallel to the *X*-axis. Therefore, the prepared specimens can be assumed to be a five-layered composite of which the magnetostrictive layer is located on the second layer.

**Figure 7:**
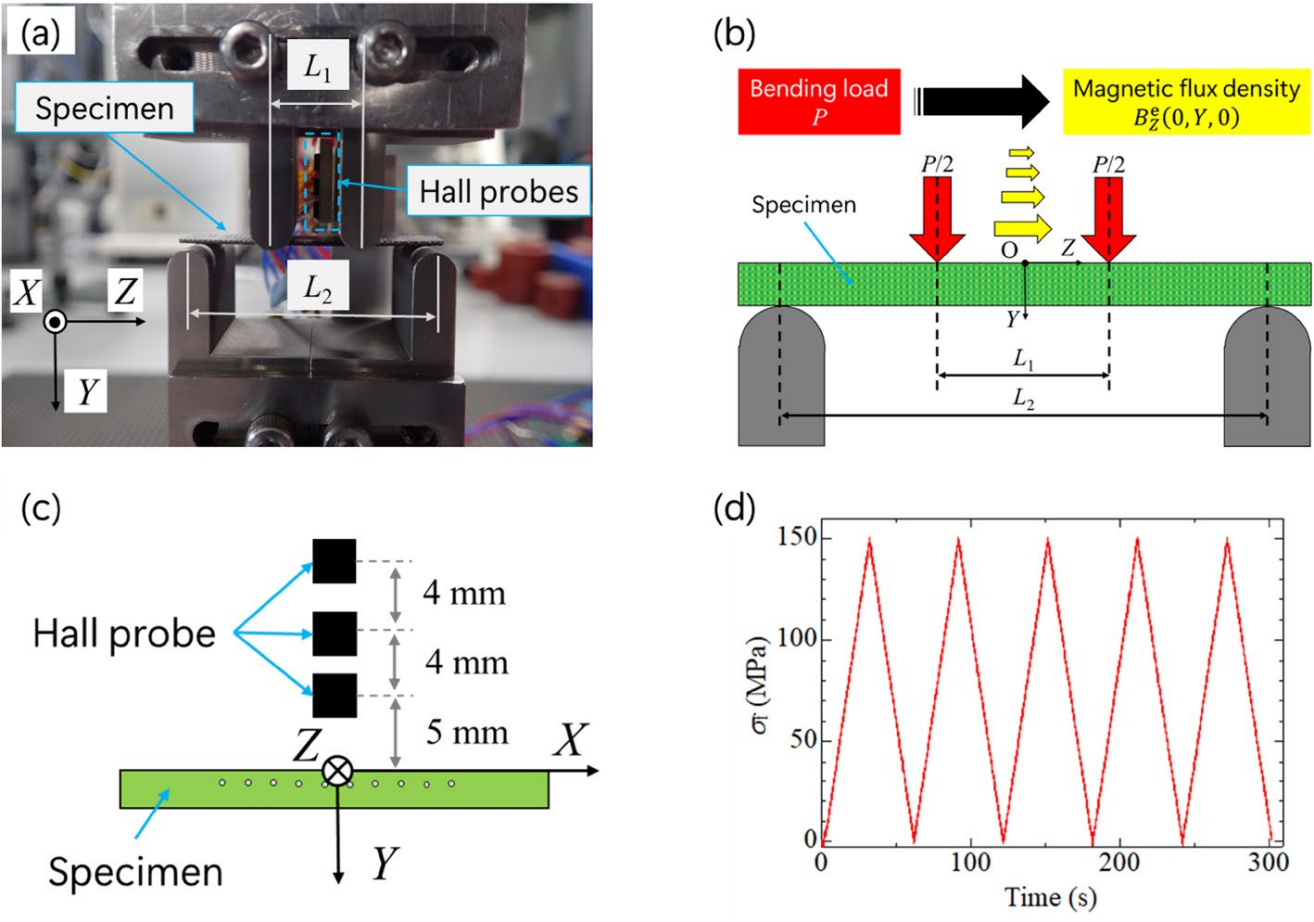
(**a**) Experimental setup of four-point bending test, (**b**) sketch of magnetic flux density distribution caused by external bending load, (**c**) the Hall probes’ position for measurement of magnetic flux density distribution, and (**d**) four-point bending test program.

Four-point bending tests were carried out using Autograph (AG-50kNXD, Shimadzu Corporation, Japan). Figure 7a shows the experimental setup of the four-point bending test. The load and support spans were *L*_1_ = 12 and *L*_2_ = 34 mm, respectively. Three Hall probes (HG-302C, Asahi Kasei Microdevices Corporation, Japan) were positioned above the specimens to measure the magnetic flux density change $B_{Z}^{{\text{e}}}$ in the longitudinal (*Z*-)direction as shown in Fig. 7b. The distance between the specimen surface and the center of the Hall probe, |*Y*|, was 5, 9, and 13 mm, respectively (shown in Fig. 7c). Figure 7d shows a four-point bending test program. The specimens were loaded under stress control at a rate of 5 MPa/s, and the maximum load was approximately 150 MPa. Four-point bending tests were carried out to evaluate the reproducibility of inverse magnetostrictive responses corresponding on bending load without a bias magnetic field. All the analog signals, which are load, *P*, and load point displacement, *δ*, from Autograph, and voltage, *V*, from Hall probes were simultaneously collected by the data logger (NR-500 series, KEYENCE Corporation, Japan). The magnetic flux density, $B_{Z}^{{\text{e}}}$, was computed by multiplying the measured voltage from a Hall probe with the coefficient, 0.8 mT/mV, obtained from the data sheet of the Hall probe.

## Results and discussion

**Figure 8:**
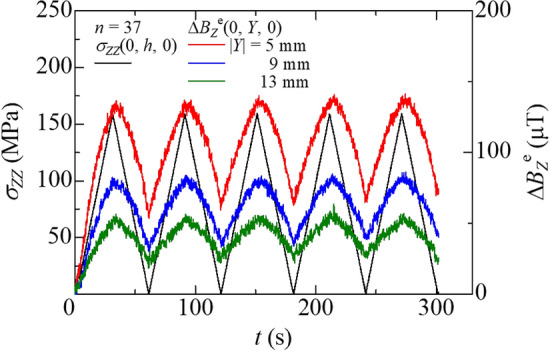
Bending stress and magnetic flux density change versus time for the specimen with 37 Fe–Co fibers.

The bending stress at the bottom surface *σ*_f_ = *σ*_*ZZ*_(0, *h*, 0) and the magnetic flux density change $B_{Z}^{{\text{e}}} \left( {0, Y,0} \right)$ are plotted in Fig. 8 as a function of the time *t* for the specimen with 37 Fe–Co fibers. The magnetic flux density’s behavior corresponded to the bending stress fluctuation. As expected, the variation of the magnetic flux density change decreased with an increase in the distance between the specimen’s surface and the Hall probe’s center. At the end of a cycle, the bending stress almost became 0 MPa; however, the magnetic flux density did not return to the initial value. This result can be understood since the residual magnetization of the Fe–Co fiber affects the behavior. The residual magnetization will be an important problem for sensor applications; however, the reproducible behavior of the magnetic flux density’s change was observed under the bending stress. The similar results were obtained for the specimens with 5, 10, and 20 Fe–Co fibers. These results indicate that Fe–Co fiber/GFRP composites can monitor bending stress. Table 2 shows the maximum change of the magnetic flux density $B_{{Z,{\text{max}}}}^{{\text{e}}}$ of all specimens. Here, the maximum change of the magnetic flux density was calculated by taking the difference between the peak value and the end of cycle value. The result of the specimen with 37 Fe–Co fibers was the largest of all specimens.

**Table 2:** Maximum change of the magnetic flux density for all specimens.

*n*	$B_{Z,\max }^{\rm{e}}$(μT)		
	|*Y*| (mm)		
	5	9	13
5	17.3	6.4	6.0
10	29.3	15.9	11.2
20	49.5	28.6	17.4
37	70.7	42.5	27.1

**Figure 9:**
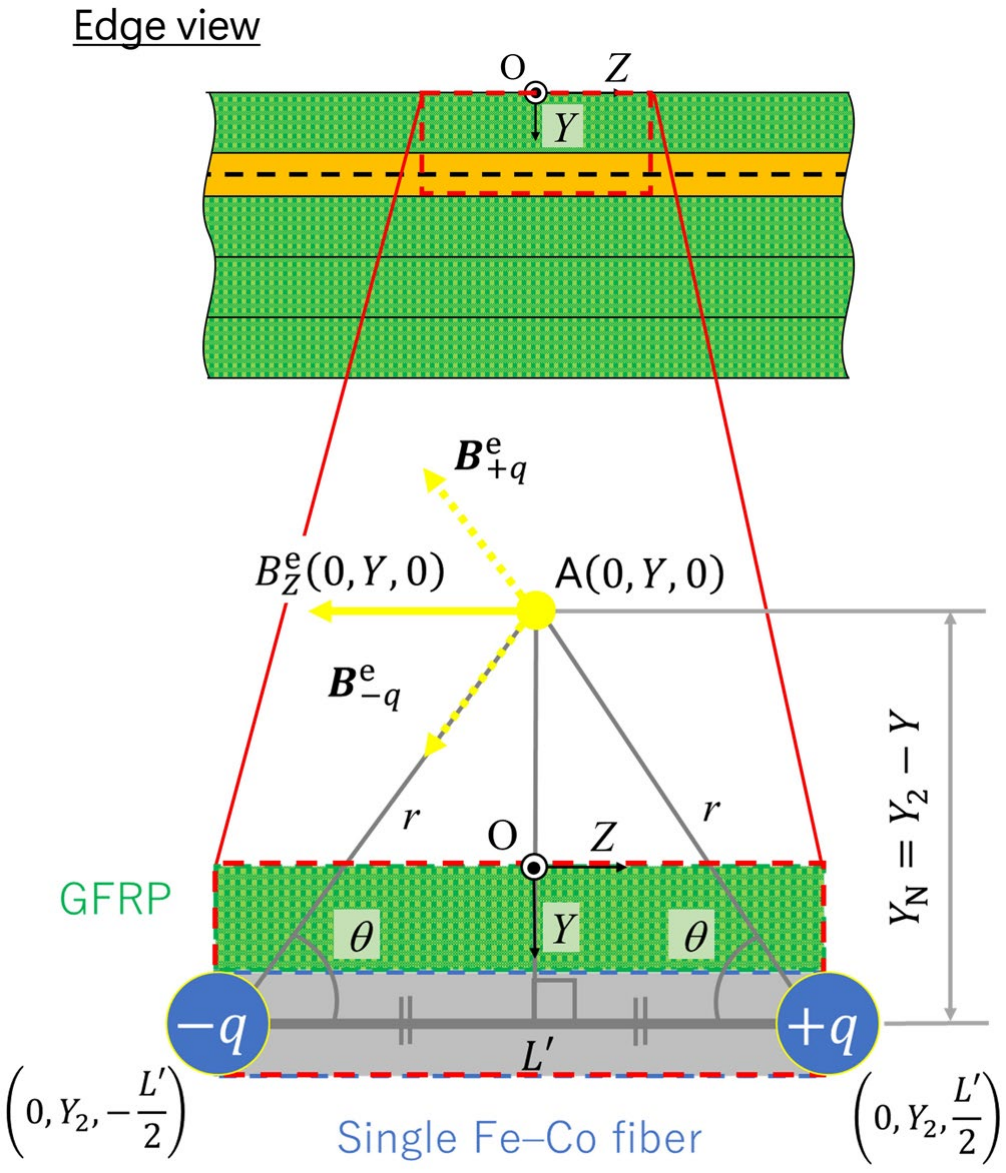
Schematic of modeled Fe–Co fiber and arbitrary point A.

**Figure 10:**
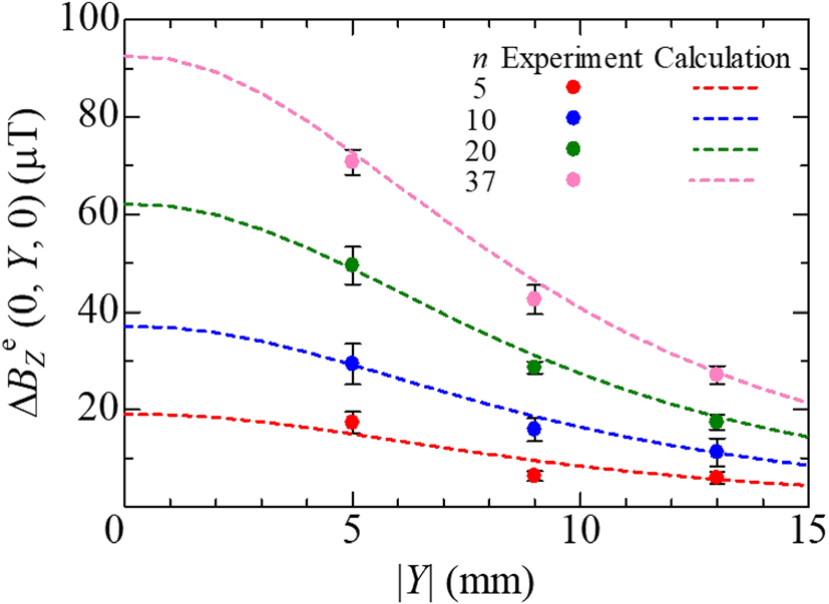
Magnetic flux density change versus the distance between the specimen surface and the Hall probe’s center.

Table 3 lists the calculated values of the magnetic flux density of Fe–Co fiber $B_{Z}^{{\text{f}}} \left( {0,Y_{2} ,0} \right) = B_{z}^{{\text{f}}} \left( {0,0,0} \right)$ by Eq. (10) and magnetic charge *q* by Eq. (6) under maximum bending stress. Here, *n* Fe–Co fibers closely spaced at the center of the magnetostrictive layer’s width were assumed as one Fe–Co fiber with length, *L*′, and cross-sectional area, *S* = π*nd*^2^/4, and the single fiber was located at the center of the magnetostrictive layer (single magnetic rod model). The magnetic charges, + *q* and − *q*, are placed at (0, *Y*_2_, *L*′/2) and (0, *Y*_2_, − *L*′/2), respectively. Figure 9 shows the schematic of a modeled single Fe–Co fiber and arbitrary point A. Figure 10 gives a plot of the magnetic flux density change in the air with the distance between the specimen surface and the Hall probe’s center showing the calculated values and the experimental data for all specimens. The dots are the average values obtained by the experimental data at |*Y*|= 5, 9, and 13 mm. The dashed lines were plotted by substituting the values in Table 3 to Eq. (10). The trend is sufficiently similar between the calculation and the experiment. This result implies that the suggested model is useful, and that the Fe–Co fiber’s magnetic flux density can be predicted during the four-point bending test by monitoring the variation of the magnetic induction around the Fe–Co fiber/GFRP composites with a Hall probe. The magnetic charges *q* in Table 3 will be discussed later.

**Table 3:** Calculated values of magnetic flux density, total cross-sectional area, and magnetic charge under maximum bending stress.

*n*	*B*_*z*_^f^ (0, *Y*_2_, 0) (mT)	*S* (× 10^−3^ mm^2^)	*q* = *B*_*z*_^f^ *S* (nWb)
5	403.4	39.27	15.84
10	393.0	78.54	30.87
20	328.8	157.1	51.65
37	264.9	290.6	76.97

**Figure 11:**
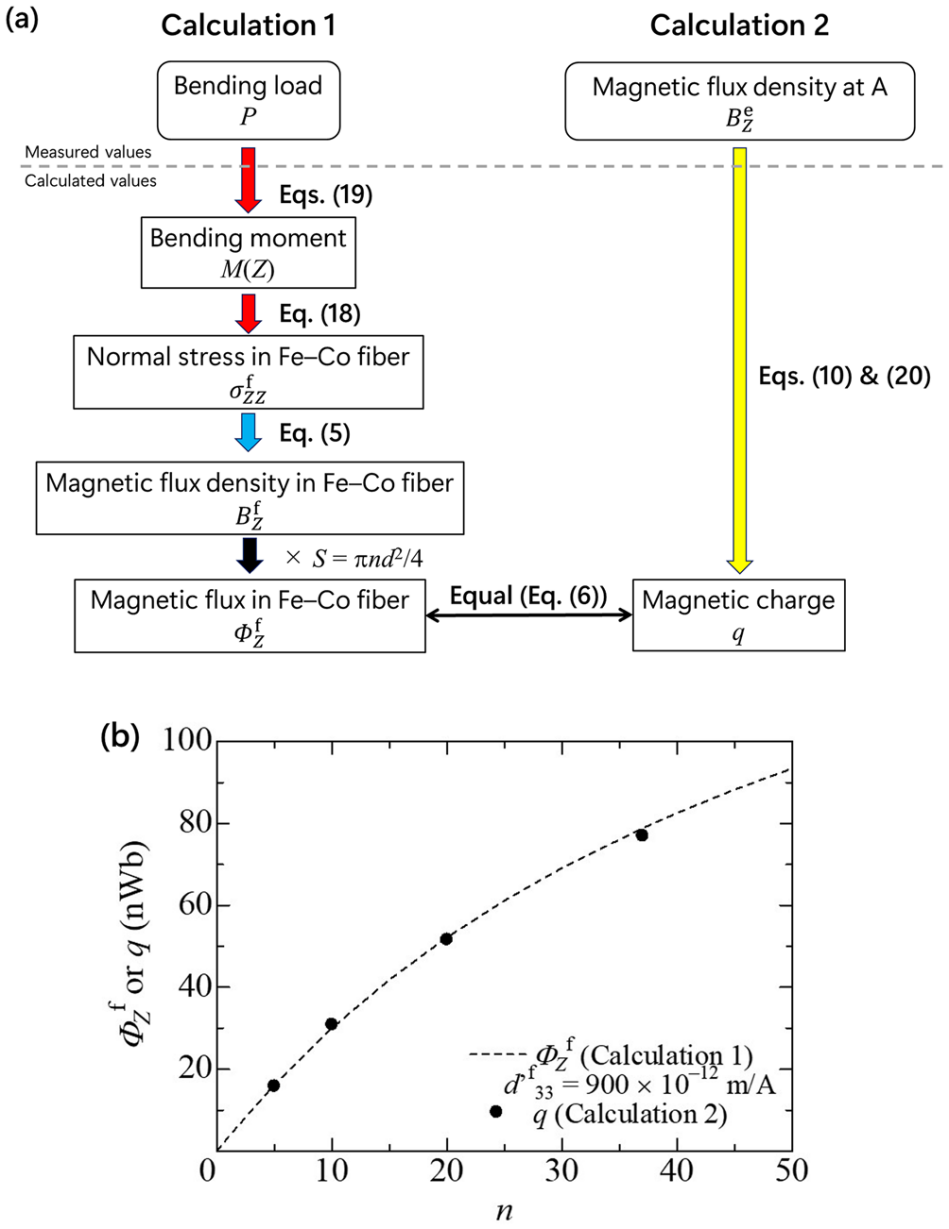
(**a**) Scheme of estimation of magnetic flux in Fe–Co fibers, and (**b**) magnetic flux versus the number of Fe–Co fibers.

Figure 11a illustrates the scheme of estimating the Fe–Co fibers’ magnetostrictive response under the bending moment from the bending load (Calculation 1) and the measured magnetic flux density (Calculation 2). Figure 11b shows the magnetic flux versus the number of Fe–Co fibers. The dashed line denotes the calculated data ${\phi_{z}^{\text{f}}}$ based on the composite beam model consisting of one magnetostrictive layer and four GFRP layers. The magnetic flux increases with increase in the number of Fe–Co fibers. The line represents the good agreement with the dots *q* from Table 3 when the magnetoelastic constant $d^{\prime\rm{f}}_{33}$ is assumed as 900 × 10^−12^ m/A. When the applied bending stress is assessed using a coil or a Hall probe, the amount of magnetic flux is important because it affects the difficulty of monitoring magnetic flux density in space. This result implies the validity of the composite beam model and single magnetic rod model to correlate the external bending load in relation to the magnetostrictive response. In other words, the analytical procedure was proposed to monitor bending stress *σ*_f_ from magnetic flux density ***B***^e^ in space, as shown in Fig. 1.

## Conclusion

This study focused on establishing the novel models to correlate an inverse magnetostrictive response and a bending stress. The Fe–Co fiber was assumed to have two magnetic charges at both ends, and the magnetic charges virtually induced the magnetic field in the air. The five-layered composite beam with four GFRP layers and one magnetostrictive layer was considered under bending. The magnetostrictive layer consisted of *n* Fe–Co fibers and an epoxy matrix. To devise the analytical procedure using the proposed models, we fabricated the Fe–Co fiber/GFRP composites and performed four-point bending tests. The stress monitoring capability was also investigated. The magnetostrictive response of the Fe–Co fiber/GFRP composites was calculated by using the composite beam and single magnetic rod models, which take into account the Villari effect. During the four-point bending tests, the magnetic flux density outside the Fe–Co fiber/GFRP composite specimen was monitored, using three Hall probes with different distances from the specimen’s surface. The magnetic flux density behavior corresponded to the bending stress fluctuation. The maximum magnetic flux density change was 70.7 mT subjected to the peak bending stress of 158 MPa. The composite beam and single magnetic rod models also predicted the distributions of the magnetic flux density around the various magnetostrictive composites with different numbers of Fe–Co fibers, which were in good agreement with the experimental results. In addition, the magnetoelastic constant was predicted, and the relationship between the bending stress (or load) and magnetic flux was successfully obtained. This study can be exploited to realize the contactless SHM technique utilizing the magnetostrictive materials because the theoretical procedure enables to predict a bending stress in a structure through monitoring magnetic flux density in the free space.

The stress monitoring method is an important step for the realization of damage/fracture sensing. In the future work, the relationship between an inverse magnetostrictive response and the fracture behavior of Fe–Co fiber/GFRP composites will be discussed in detail.

## Data Availability

The datasets used and/or analyzed during the current study are available from the corresponding author on reasonable request.
